# SARS-CoV-2 genomic surveillance in Taiwan revealed novel ORF8-deletion mutant and clade possibly associated with infections in Middle East

**DOI:** 10.1080/22221751.2020.1782271

**Published:** 2020-07-03

**Authors:** Yu-Nong Gong, Kuo-Chien Tsao, Mei-Jen Hsiao, Chung-Guei Huang, Peng-Nien Huang, Po-Wei Huang, Kuo-Ming Lee, Yi-Chun Liu, Shu-Li Yang, Rei-Lin Kuo, Kuan-Fu Chen, Yen-Chin Liu, Sheng-Yu Huang, Hsing-I. Huang, Ming-Tsan Liu, Ji-Rong Yang, Cheng-Hsun Chiu, Cheng-Ta Yang, Guang-Wu Chen, Shin-Ru Shih

**Affiliations:** a Research Center for Emerging Viral Infections, College of Medicine, Chang Gung University, Taoyuan, Taiwan; b Department of Laboratory Medicine, Linkou Chang Gung Memorial Hospital, Taoyuan, Taiwan; c Department of Medical Biotechnology and Laboratory Science, College of Medicine, Chang Gung University, Taoyuan, Taiwan; d Division of Infectious Diseases, Department of Pediatrics, Linkou Chang Gung Memorial Hospital, Taoyuan, Taiwan; e Graduate Institute of Biomedical Sciences, College of Medicine, Chang Gung University, Taoyuan, Taiwan; f Division of Asthma, Allergy, and Rheumatology, Department of Pediatrics, Linkou Chang Gung Memorial Hospital, Taoyuan, Taiwan; g Department of Emergency Medicine, Chang Gung Memorial Hospital, Keelung, Taiwan; h Clinical Informatics and Medical Statistics Research Center, Chang Gung University, Taoyuan, Taiwan; i Community Medicine Research Center, Chang Gung Memorial Hospital, Keelung, Taiwan; j Department of Pediatrics, Linkou Chang Gung Memorial Hospital, Taoyuan, Taiwan; k Centers for Disease Control, Taipei, Taiwan; l Division of Pediatric Infectious Diseases, Department of Pediatrics, Chang Gung Memorial Hospital, Chang Gung University College of Medicine, Taoyuan, Taiwan; m Molecular Infectious Disease Research Center, Chang Gung Memorial Hospital, Chang Gung University College of Medicine, Taoyuan, Taiwan; n Department of Respiratory Therapy, College of Medicine, Chang Gung University, Taoyuan, Taiwan; o Department of Thoracic Medicine, Linkou Chang Gung Memorial Hospital, Taoyuan, Taiwan; p Department of Computer Science and Information Engineering, School of Electrical and Computer Engineering, College of Engineering, Chang Gung University, Taoyuan, Taiwan; q Research Center for Chinese Herbal Medicine, Research Center for Food and Cosmetic Safety, and Graduate Institute of Health Industry Technology, College of Human Ecology, Chang Gung University of Science and Technology, Taoyuan, Taiwan

**Keywords:** COVID-19, SARS-CoV-2, genome sequencing, Phylogeny, ORF8 deletion

## Abstract

Taiwan experienced two waves of imported infections with Coronavirus Disease 2019 (COVID-19). This study aimed at investigating the genomic variation of severe acute respiratory syndrome coronavirus 2 (SARS-CoV-2) in Taiwan and compared their evolutionary trajectories with the global strains. We performed culture and full-genome sequencing of SARS-CoV-2 strains followed by phylogenetic analysis. A 382-nucleotides deletion in open reading frame 8 (ORF8) was found in a Taiwanese strain isolated from a patient on February 4, 2020 who had a travel history to Wuhan. Patients in the first wave also included several sporadic, local transmission cases. Genomes of 5 strains sequenced from clustered infections were classified into a new clade with ORF1ab-V378I mutation, in addition to 3 dominant clades ORF8-L84S, ORF3a-G251V and S-D614G. This highlighted clade also included some strains isolated from patients who had a travel history to Turkey and Iran. The second wave mostly resulted from patients who had a travel history to Europe and Americas. All Taiwanese viruses were classified into various clades. Genomic surveillance of SARS-CoV-2 in Taiwan revealed a new ORF8-deletion mutant and a virus clade that may be associated with infections in the Middle East, which contributed to a better understanding of the global SARS-CoV-2 transmission dynamics.

## Introduction

Coronaviruses (CoVs) are classified into four genera, including *Alphacoronavirus*, *Betacoronavirus*, *Gammacoronavirus*, and *Deltacoronavirus* [1]. Prior to 2019, six CoVs were known to infect human, including human CoV 229E and NL63 belonging to the genera *Alphacoronavirus*, and human CoV OC43, HKU1, Severe Acute Respiratory Syndrome-related CoV (SARSr-CoV), and Middle East Respiratory Syndrome CoV (MERS-CoV) belonging to the genera *Betacoronavirus* [2–5]. The seventh and novel coronavirus emerged from Wuhan, Hubei province in China, in December 2019 [6]. This virus belongs to the genera *Betacoronavirus* and has been designated as Severe Acute Respiratory Syndrome Coronavirus 2 (SARS-CoV-2), and the disease is named as Coronavirus Disease 2019 (COVID-19) [7]. The World Health Organization declared this disease a pandemic on March 11, 2020.

As of April 14, 2020, the outbreak of COVID-19 has resulted in 1,844,863 confirmed cases and 117,021 deaths worldwide [8], among which 393 confirmed cases and 6 deaths were reported in Taiwan [9]. With rapidly increasing number of infections, the Global Initiative on Sharing All Influenza Data (GISAID) [10] provides a platform for sharing SARS-CoV-2 sequences and their metadata. Three major clades, including clade G (with G variant at position 614 within the spike protein, as S-D614G), clade S (ORF8-L84S), and clade V (ORF3a-G251V), were designated by the GISAID (https://www.gisaid.org/hcov-19-analysis-update/). However, the imbalance of genome information contributed to this platform could prevent us from comprehensively understand the viral transmission and epidemiology of this pandemic. In particular that none or very few sequences have been reported from some countries. With more virus genomes become available, we would be able to better address the diversification and evolution of this virus.

There have been two waves of COVID-19 cases in Taiwan. The first occurred from late January to the end of February, with most infections originating from China either by Chinese tourists or Taiwanese businessmen returning to Taiwan from China for celebrating Chinese New Year. The second and bigger wave started in early March, during which the infections were largely caused by Taiwanese tourists, business travellers, or students returning from other countries. Although most of these cases were traced to their foreign origins, some small and clustered infections were suspected to have been resulted from local transmission.

In this study, we performed culture and full-genome sequencing of SARS-CoV-2 isolates, and further investigated the viral genome polymorphism within individual human clinical specimens through their metagenomic data. We compared the genomes of Taiwanese strains to those of global strains to describe their genetic variations and evolutionary trajectory. Strikingly, an open reading frame 8 (ORF8) deletion was found in one Taiwanese strain. Moreover, a highlighted clade with ORF1ab-V378I mutation was observed in addition to the three major clades designated by the GISAID. Interestingly, included in this clade were some strains isolated from patients with travel history to Turkey and Iran, where few viral genomes have yet been reported in the Middle East region.

## Materials and Methods

### Ethics statement

This study was approved by the Institutional Review Board of Chang Gung Medical Foundation, Linkou Medical Center, Taoyuan, Taiwan (approval no. 202000468B0B1).

### Specimen collection

SARS-CoV-2 infection in patients was confirmed by real-time reverse-transcriptase–polymerase chain reaction (RT-PCR) according to the guidelines of the Taiwan Centers for Disease Control (CDC; https://www.cdc.gov.tw/En) [11]. All nasopharyngeal swab, throat swab, and sputum samples were maintained in viral transport medium for further analysis.

### Cell culture and virus Isolation

Vero-E6 (ATCC, Manassas, VA, USA), MK-2 (ATCC), and Calu-3 (ATCC) cells were maintained in Minimum Essential Medium (MEM, Thermo Fisher Scientific, Waltham, MA, USA) supplemented with 10% fetal bovine serum and 1x penicillin-streptomycin at 37°C in the presence of 5% CO_2_. To isolate the virus, all procedures were performed in accordance with the laboratory biosafety guidelines of the Taiwan CDC in a biosafety level-3 facility. Cells grown to 80–90% confluency in standard screw-up culture tubes (16 × 125 mm) were inoculated with 500 μL of the virus solution. The virus solution was prepared by diluting 100 μL of the specimen samples with 1.5 mL of sample pretreatment medium consisting of MEM, 2% fetal bovine serum, and 2x penicillin-streptomycin solution, followed by incubation at 37°C for 1 h. Virus absorption process was allowed at 37°C for 1 h, after which 2 mL of the virus culture medium composed of MEM, 2% fetal bovine serum, and 1x penicillin-streptomycin solution was added, and the culture mixture was maintained at 37°C. Infected cells were observed daily to determine the cytopathic effect of the virus on the cells. Additionally, RT-PCR analysis using the RNA extracted from part of the culture supernatant every 2 days after inoculation was performed to monitor viral growth. We continuously observed the infected cells until the cytopathic effect was noticed in more than 75% of the cells, after which the culture supernatant was harvested and stored at –70°C. Viral titer was determined by Vero-E6 cells with medium consisting of DMEM, 2% fetal bovine serum and 0.4% agarose, followed by incubation at 37°C for 3 days.

### Whole-Genome sequencing

Twenty samples (CGMH-CGU No. 1–20) were collected (Table 1). RNA was extracted either from the culture supernatant or directly from the specimens using a QIAmp viral RNA mini Kit (Qiagen, Hilden, Germany) according to the manufacturer’s instructions, except that the carrier RNA was replaced with linear acrylamide (Thermo Fisher Scientific) as the co-precipitant. The amount of viral RNA was evaluated by quantitative RT-PCR to examine the Ct value of the viral E gene (Table 1). For RNAs showing a high Ct value, we used the Ovation RNA-Seq System V2 (Nugen Technologies, San Carlos, CA, USA) to synthesize cDNA that was further processed into a library using the Celero DNA-Seq System (Nugen Technologies). Other samples with lower Ct values were used for library preparation by using the Trio RNA-Seq kit (Nugen Technologies). Next-generation sequencing (NGS) was performed on an Illumina MiSeq System (San Diego, CA, USA) with paired-end reads. More than 0.75 and 2.5 Gb of raw data per sample were generated from viral isolates and clinical specimens, respectively.

**Table 1.** Specimen collection, culture, and sequencing.

CGMH-CGU ID / Strain name	Accession number	Collection date	Viral culture (day)	Source* (Ct value of E gene)	Coverage and avg. depth of SARS-CoV-2
1	EPI_ISL_411915	2020-01-25	–	SP (17.01)	99.9%; 1157.4
2	EPI_ISL_417518	2020-02-04	14	MK2 (10.0)	100.0%; 5891.8
3	EPI_ISL_415741	2020-02-26	10	MK2 (14.25)	100.0%; 18,300.8
4	EPI_ISL_415742	2020-02-27	4	Vero E6 (26.15)	99.2%; 449.6
5	EPI_ISL_415743	2020-02-27	4	MK2 (12.78)	100.0%; 26,521.5
6	EPI_ISL_417519	2020-03-05	5	MK2 (12.82)	100.0%; 13,718.5
7	EPI_ISL_417520	2020-03-09	-	SP (22.98)	100.0%; 53.1
8	EPI_ISL_417521	2020-03-10	-	NP (23.18)	100.0%; 11,499.4
9	EPI_ISL_417522	2020-03-13	3	MK2 (10.89)	100.0%; 30,044.7
10	EPI_ISL_417523	2020-03-13	3	MK2 (10.45)	100.0%; 29,572.7
11	EPI_ISL_417524	2020-03-14	3	Vero E6 (11.08)	100.0%; 24,326.9
12	EPI_ISL_417525	2020-03-14	3	MK2 (10.11)	100.0%; 34,422.0
13	EPI_ISL_424969	2020-03-17	7	MK2 (9.12)	100.0%;10,227.7
14	EPI_ISL_424970	2020-03-17	7	MK2 (9.92)	100.0%;15,863.1
15	EPI_ISL_424971	2020-03-17	7	MK2 (10.94)	100.0%;14,292.3
16	EPI_ISL_424972	2020-03-16	7	MK2 (12.37)	100.0%;11,611.7
17	EPI_ISL_424973	2020-03-17	7	MK2 (11.57)	100.0%;21,393.0
18	EPI_ISL_424974	2020-03-18	7	MK2 (10.02)	100.0%;15,382.4
19	EPI_ISL_424975	2020-03-18	7	Vero E6 (11.93)	100.0%;9395.7
20	EPI_ISL_424978	2020-03-18	7	Vero E6 (11.50)	100.0%;13,364.3
Taiwan/2	EPI_ISL_406031	2020-01-23	–	–	–
Taiwan/3	EPI_ISL_411926	2020-01-24	–	–	–
Taiwan/125	EPI_ISL_420082	2020-03-19	–	–	–
Taiwan/128	EPI_ISL_420083	2020-03-18	–	–	–
Taiwan/170	EPI_ISL_420084	2020-03-21	–	–	–
Taiwan/107	EPI_ISL_420085	2020-03-20	–	–	–

*Sources are sputum (SP) and nasopharyngeal swab (NP) specimens, or supernatants of cultured MK2 and Vero E6 cells.

### Next-generation sequencing data analysis pipeline

We first trimmed the raw data by removing low-quality and short reads using Trimmomatic (version 0.39) [12]. Next, quality reads were mapped to the human reference genome to remove host sequences using HISAT2 (version 2.1.0) [13]. SPAdes (version 3.14.0) [14] was then used to perform *de novo* assembly for constructing contig sequences, and BLASTN was used to query the assembled contigs by searching the nucleotide sequence (NT) database of the National Center for Biotechnology Information (NCBI). Viral and bacterial candidates were identified using the top reported BLASTN hits for each of the queried contig sequences, followed by mapping NGS reads onto these candidates to reveal the metagenome. To specifically determine the SARS-CoV-2 genome of interest, on the other hand, we used a reference strain Wuhan-1 (accession number MN908947.3) as the mapping template and used an iterative mapping approach [15] to increase the read depth and coverage. Reads mapping to the obtained viral genome from iterative mapping was used for assembling again by SPAdes to re-confirm the genomic context. Distributions of read coverages were generated by R package ggplot2 [16].

### Phylogenetic and sequence analysis

Out of the 20 whole genomes assembled in this study, 17 were obtained from the virus isolates and three were directly from specimens (Table 1; with additional six genomes provided by Taiwan CDC). In total, genomic data of 26 strains were deposited in the GISAID with accession numbers also given in Table 1. We also downloaded all complete and high-coverage genomes of SARS-CoV-2 from GISAID as of March 14, 2020 and obtained 332 sequences after removing those with sequence gaps or ambiguous nucleotides. The reference strain MN908947.3 from GenBank (NCBI) was also included. As the result, a total of 359 sequences were aligned using MAFFT (version 7.427) [17]. The phylogenetic tree was inferred using RAxML (version 8.2.12) [18] under the GTRGAMMA model with 1000 bootstrap replicates to investigate the genomic relationships between virus strains.

### RT-PCR and viral growth analyses for the ORF8-deletion mutant

The ORF8 deletion found in one Taiwanese CGMH-CGU-02 isolate was verified by NGS directly from the specimen, as well as amplifying and re-sequencing the concerned region using Sanger method. Briefly, cDNA preparation was also performed using the MMLV Reverse Transcription kit (Protech, Taiwan) according to the manufacturer’s instructions. The primer sequences used to amplify ORF8 containing region or E gene are as follows: SARS-CoV-2-E-For: 5′- ATGTACTCATTCGTTTCGGAAGAGAC-3′, SARS-CoV-2-E-Rev: 5′-TTAGACCAGAAGATCAGGAACTCTAG-3′, SARS-CoV-2-27760-For: 5′-TTGAACTTTCATTAATTGACTTCTATTTGTG-3′, and SARS-CoV-2-N-Rev: 5′-TTAGGCCTGAGTTGAGTCAGCACTGCTC-3′.

To examine the effect of ORF8 deletion on viral growth, 4 × 10^5^ Calu-3 cells grown in a 12-well plate were infected with either CGMH-CGU-01 or CGMH-CGU-02 at an MOI of 0.05. Total RNA was extracted from infected cells using TRIzol reagent (Thermo Fisher Scientific) at 24 and 48 h post-infection, according to the manufacturer’s instructions. RT-PCR was performed to examine the viral growth, and primers and probes used were described by Corman et al. [11]. Genome copies were converted from Ct by using in vitro transcribed RNA of RNA-dependent RNA polymerase (RdRp, also named nsp12) and Envelope (E) genes as the standard RNA. Normalization was based on the Ct of β-actin using primers as follows: β-actin-For: 5′- CTACAATGAGCTGCGTGTGG-3′; β-actin-Rev: 5′-GCTCATTGCCAATGGTGATG-3′.

## Results

### ORF8 deletion revealed by NGS data analysis

**Figure 1.**
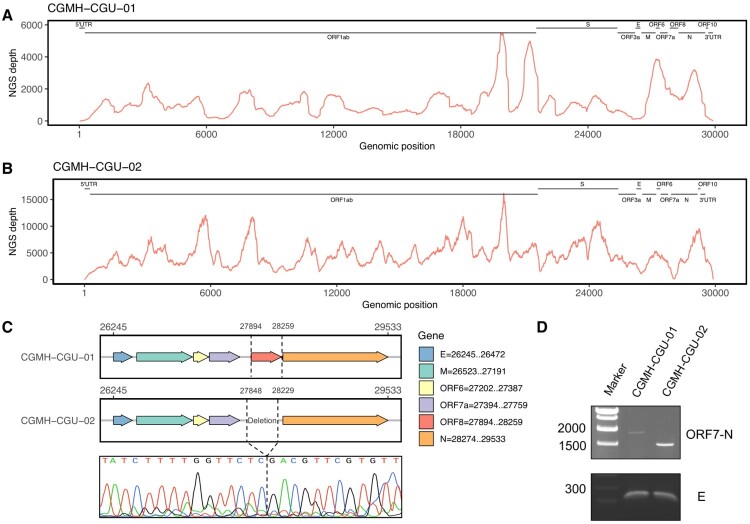
ORF8 deletion in SARS-CoV-2 genome. (A and B) NGS depths of CGMH-CGU-01 and CGMH-CGU-02. (C) Genomic regions of ORF8 and deletion according to the genomic positions of reference strain Wuhan-1 (MN908947.3), and Sanger sequence of this deletion in CGMH-CGU-02. (D) ORF8 deletion was further verified by RT-PCR showing a reduced amplicon size.

Complete genomic data of 20 SARS-CoV-2 strains obtained in this study were derived from 3 specimens (CGMH-CGU-01, -07, and -08) and 17 isolates (-02 to -06 and -09 to -20), and have been uploaded in GISAID. Table 1 shows their NGS coverage and depth. The average depths for all the 17 isolates were greater than 9000X, except that CGMH-CGU-02 and -04 have 5892X and 450X respectively. Among the three genomes derived directly from the specimens, one (CGMH-CGU-08) has comparably high depth as virus isolate genomes did, whereas two (CGMH-CGU-01 and -07) showed a lower depth of 1157.4 and 53.1, respectively. Table 1 also includes the 6 strains previously submitted by Taiwan CDC. Figure 1A shows the NGS coverage and depth distribution of CGMH-CGU-01, which has an identical genome as the Wuhan-1 strain (accession number MN908947.3). Notably, we detected a deletion in a 382-nucleotide (nt) sequence at the genomic positions 27,848–28,229 in one Taiwanese genome (CGMH-CGU-02) as shown in Figure 1B. This deletion mostly overlaps with the ORF8 of 366-nt (27,894–28,259) according to the CGMH-CGU-01 or Wuhan-1 strain (Figure 1C). Since the CGMH-CGU-02 genome was determined from a virus isolate, we further performed NGS using a specimen directly from the same patient for verifying this deletion. Reads yielding the same 382-nt deletion were confirmed in the original specimen, although only a partial genome was assembled with a coverage and average depth of 80.0% and 4.2, respectively. RT-PCR was also performed to verify the NGS data, wherein we designed the primers pairing with the end of ORF7 and N gene, respectively. Sanger sequence (bottom of Figure 1C) shows the evidence of this ORF8 deletion, which reduced the amplicon size from 1774 to 1329 nt (Figure 1D, CGMH-CGU-02).

CGMH-CGU-02 was isolated from a patient with a travel history to Wuhan and who returned to Taiwan on February 3, 2020. Clinical specimen from this patient was collected on February 4, indicating that this ORF8-deletion has already occurred in China. Four nucleotide substitutions were found in this strain when compared to the genome of the Wuhan-1 strain. Two were located at the ORF1ab position 8517 changing from C to T (as C8517T) and position 16577 from A to G (A16577G). The latter was a non-synonymous mutation resulting in an amino acid change at position 5526 from K to R (K5526R). The other two mutations in CGMH-CGU-02 were also non-synonymous, located in the S gene at C145T (H49Y) and C2651T (S884F). Nevertheless, 3 strains isolated from Singapore have shown the same 382-nt ORF8 deletion as of April 14, 2020. Mutations from these ORF8-deletion genomes by comparing to the reference strain were summarized in Table 2. One strain hCoV-19/Singapore/14/2020 showed only one synonymous mutation ORF1ab-C8517T, which was also found in CGMH-CGU-02. The other two (hCoV-19/Singapore/12/2020 and hCoV-19/Singapore/13/2020) each additionally showed two different non-synonymous mutations S-T2449C (F817L) and ORF3a-C176A (A59D), and ORF1ab-T17459C (V5820A) and N-C595T (P199S), respectively. These Singapore strains were collected from clustered infections from February 13–18, 2020, and apparently had different mutations compared with those in CGMH-CGU-02.

**Table 2.** Genomic mutations of Taiwanese and Singapore strains with the ORF8-deletion.

Strain name	Nucleotide (amino acid) mutation
CGMH-CGU-02	ORF1ab-C8517T, ORF1ab-A16577G (K5526R), S-C145T (H49Y), S-C2651T (S884F)
hCoV-19/Singapore/12/2020	ORF1ab-C8517T, S-T2449C (F817L), ORF3a-C176A (A59D)
hCoV-19/Singapore/13/2020	ORF1ab-C8517T, ORF1ab-T17459C (V5820A), N-C595T (P199S)
hCoV-19/Singapore/14/2020	ORF1ab-C8517T

*Nucleotide or amino acid position was based on gene position of the reference strain (Wuhan-1).

Since we successfully isolated the strain containing ORF8-deletion, we further investigated the impact of this deletion on viral replication. Human Calu-3 cells were infected with CGMH-CGU-01 and -02 at an MOI of 0.05 for 24 and 48 h. We measured RNA expression levels of RdRp and E gene using RT-qPCR, and found no significant difference in RdRp and in E gene with CGMH-CGU-02 infected cells at 48 h compared with CGMH-CGU-01 (Supplementary Figure S1), indicating that the ORF8 deletion does not affect viral RNA replication in cultured human cells.

### Co-existence with Haemophilus parainfluenzae

Three out of 20 CGMH-CGU samples (-01, -07, and -08) were obtained from clinical specimens. *Haemophilus parainfluenzae* (accession number CP035368.2, a genome that is 2,067,650-bp long) was the dominant population in metagenomic contigs of CGMH-CGU-07 and -08, having a read coverage of 62.6% and 62.5% and an average depth of 53.5 and 53.5, respectively. On the other hand, metagenomic reads of CGMH-CGU-01 mapping to the same bacterial genome resulted in a less yet still sizable coverage of 21.6% and an average depth of 75.4. Particularly, some peaked NGS depths were found approaching or were over 20,000 (Supplementary Figure S2), covering 16S (approximately 1550-nt long) or 23S (2900-nt) ribosomal RNA, signalling the co-existence of *Haemophilus parainfluenzae* in these 3 Taiwanese COVID-19 patients.

### Phylogenetic tree of Taiwanese and global strains

**Figure 2.**
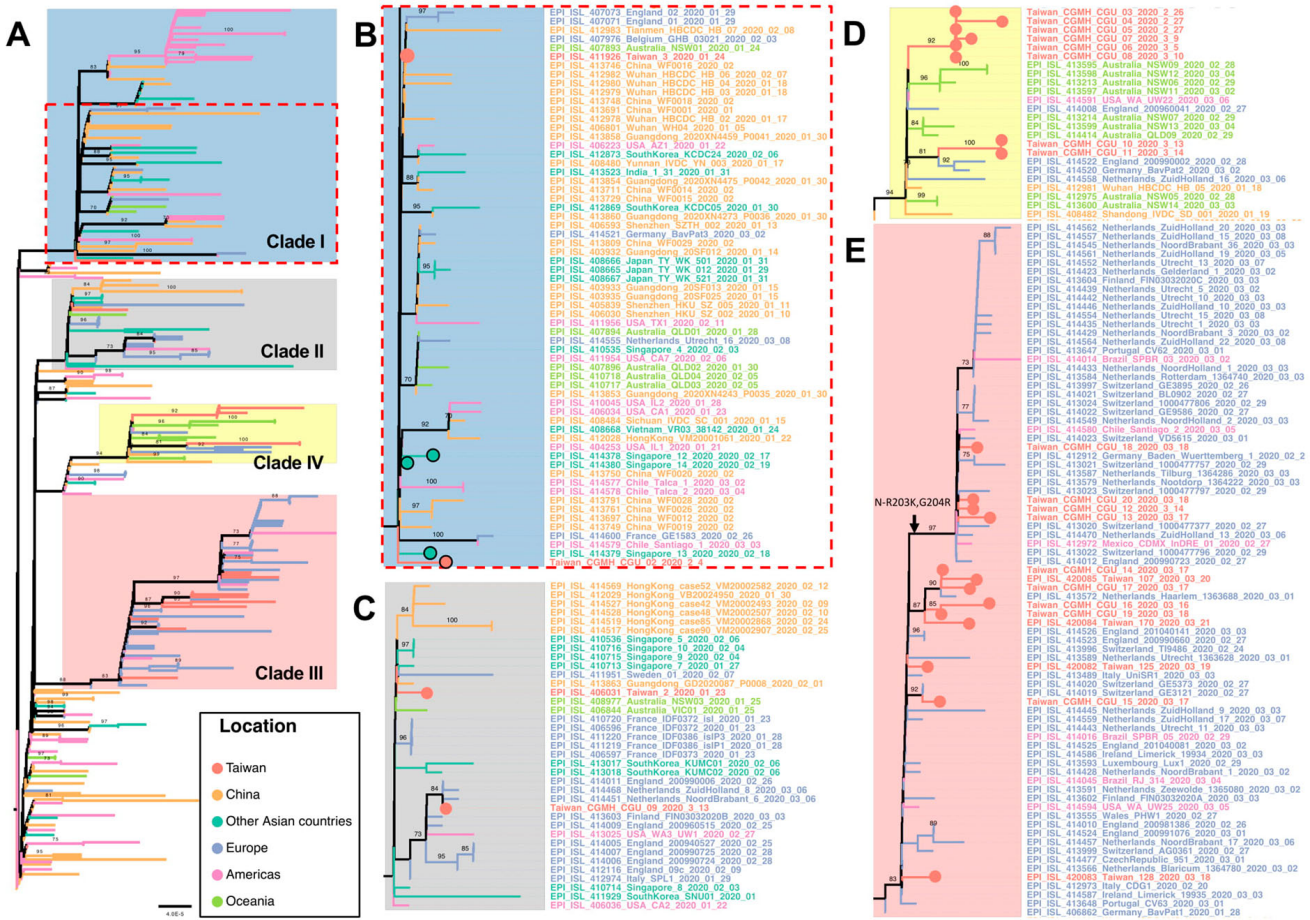
Phylogenetic tree of Taiwanese and global strains. (A) Phylogenetic analysis was performed using the maximum likelihood approach. Significant bootstrap support values greater than 70% are shown. Strains isolated from different locations and clades with specific variations are marked in different colours. Taiwanese strains are located in 4 different clades I, II, III and IV, including (B) and clade I with ORF8-L84S, (C) clade II with ORF3a-G251V, (D) clade IV with ORF1ab-V378I, and (E) clade III with S-D614G. In addition, CGMH-CGU-02 and three Singapore strains with the ORF8-deletion mutation in clade I were marked by light red and cyan solid circle, respectively.

**Figure 3.**
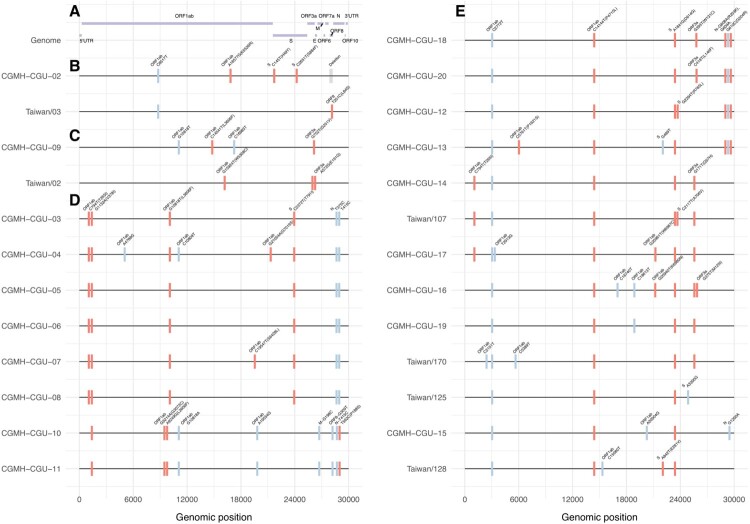
Nucleotide and amino acid variations in SARS-CoV-2 genomes. (A) SARS-CoV-2 genome is illustrated. CGMH-CGU-01 genome was identical to the reference genome, and nucleotide and amino acid variations in the SARS-CoV-2 genomes from the other 25 Taiwanese strains are shown. In line with the phylogenetic clade designation in sub-figures 2B to 2E, mutations in these 25 Taiwanese genomes are shown from (B to E), respectively. Synonymous and non-synonymous mutations were marked by blue and red bars, respectively. Amino acid changes are annotated in parentheses. ORF8 deletion in CGMH-CGU-02 genome is marked in gray.

Taiwan experienced two waves of imported cases, first from China in January to late February, followed another wave from other countries starting in early March. The phylogenetic tree of Taiwanese and global strains was constructed to infer the evolutionary relationships among the strains under consideration (Figure 2). To better illustrate phylogenetic clades, we designated and numbered yellow clade as IV harbouring ORF1ab-V378I mutation in this study and three others (blue clade I of ORF8-L84S, gray clade II of ORF3a-G251V, and pink clade III of S-D614G) identified based on previous GISAID annotations of clades S, V, and G, respectively. Figure 3 supports Figure 2 in further illustrating the SARS-CoV-2 genome variations in each of the phylogenetic clades from the 26 investigated Taiwanese genomes.

In the first wave, most Taiwanese strains identified originated from China. CGMH-CGU-01 was isolated on January 25, 2020 from a patient with a travel history to Wuhan, which has been included at the root of Figure 2 (bottom-left) together with Chinese strains in the early stage of the outbreak. Two first-wave strains (CGMH-CGU-02 and Taiwan/3) were grouped in clade I (Figure 2B). Although the CGMH-CGU-02 strain did not carry the ORF8-L84S mutation due to the aforementioned ORF8 deletion, it shared the ORF1ab-C8517T mutation with Taiwan/2 and other strains in this clade (Figures 2B and 3B). Two Taiwanese strains (Taiwan/2 from the first wave and CGMH-CGU-09 from the second wave) were grouped in clade II (Figure 2C). CGMH-CGU-09 was isolated from a patient who had a travel history to Germany, and has been clustered with other European strains. In addition to the signature mutation ORF3a-G251V of clade II, CGMH-CGU-09 also has 3 more mutations in ORF1ab (Figure 3C).

### Locally clustered and global transmission in the new ORF1ab-V378I clade

Viral genomes of CGMH-CGU-03 to -07 were determined from clustered infection samples (-03 was the index patient), together with -08 (a case originating from the United Kingdom), and -10 and -11 (having travel history to Turkey) were shown in clade IV of ORF1ab-V378I (Figure 2D). Five Australian strains (NSW05, NSW06, NSW11, NSW12, and NSW13) [19] and one German strain (BavPat2, according to the metadata from the GISAID) from patients having a travel history to Iran were also grouped in this clade. While the two earliest sequences classified in clade IV were dated mid-January and originated in Wuhan and Shandong in China, respectively, CGMH-CGU-03 was obtained from a patient who had no travel history and the specimen was collected nearly 6 weeks after the two Chinese isolates. All the other viruses in this clade were also dated after February 26. This long time gap in the identification of the two Chinese strains isolated in mid-January suggests that it is unlikely that the later strains were directly linked to the Wuhan strains. Some patients (e.g. CGMH-CGU-10 and -11) infected with virus strains belonging to this clade had a travel history to Middle East, suggesting a possible transmission route via these countries for the occurrence of clustered infections in Taiwan.

COVID-19 can be transmitted via close contact with infected patients. Regardless of whether the infected individuals are symptomatic, their family members and co-workers are at risk of infection. Viral genomes CGMH-CGU-4 to -7 were obtained from patients who had made contact with an index patient who was infected with the CGMH-CGU-03 strain. CGMH-CGU-03, -05, and -06 genomes were identical and served as the baseline for assessing pairwise genetic distances to the other two cluster members. CGMH-CGU-04 was found to have 3 ORF1ab nucleotide substitutions (A4788G, C10809T, and G21055A); the third position was a non-synonymous change with a G7019S amino acid substitution (Figure 3D). On the other hand, CGMH-CGU-07 showed only one non-synonymous substitution from the baseline at a different ORF1ab position C19547T (S6248L) (Figure 3D). These results suggest that only up to 4 nucleotide changes occurred between CGMH-CGU-04 and -07 genomes within these five Taiwanese clustered infection cases.

### Second wave initiated from infections in European countries

Taiwanese genomes in the second wave were majorly located in clade III (Figure 2E), including CGMH-CGU-12 to -20, Taiwan/107, Taiwan/125, Taiwan/128 and Taiwan/170. These strains were isolated from patients who had a travel history to European countries, except CGMH-CGU-14, -16, and -19, which is in line with the observation that many European strains were grouped to this clade. In addition to the signature mutation S-D614G annotated by GISAID in clade III, all the Taiwanese genomes shared two ORF1ab mutations C2772T and C14144T (P4715L) (Figure 3E). The most divergent genomes among the Taiwanese cases in this clade were CGMH-CGU-13 and -16, each with 8 nucleotide substitutions (resulting in 5 amino acid changes) in the coding region compared to the reference. We further found 4 Taiwanese strains (CGMH-CGU-12, -13, -18, and -20, top four in Figure 3E) shared the same N gene mutations R203K and G204R resulting from 3 nucleotide mutations G608A, G609A, and G610C.

Taken together, Figure 2(B–E) show that Taiwanese isolates were distributed in distinct lineages, indicating that no single dominant strain has been circulating in Taiwan. Figure 3 shows how all the 26 Taiwanese genomes were different from Wuhan-1 (except CGMH-CGU-01 which was identical to Wuhan-1), among which the most divergent strain was CGMH-CGU-04 that showed 9 nucleotide changes (resulting in 5 amino acid changes) in the coding region. Moreover, 8 Taiwanese genomes (including 5 determined from samples obtained from clustered infection cases in the first wave) exhibited the mutation ORF1ab-V378I that was not mentioned before; these strains have now been included in the new genetic clade IV (Figure 2D) along with some foreign strains identified from patients who had a travel history to Europe and the Middle East.

## Discussion

Timely sharing of full genomic data of SASR-CoV-2 strains with the required chronological information pertaining to different geographical locations is important for monitoring the genetic changes in the virus that may be associated with viral spreading and clinical manifestations. Clade IV featuring the ORF1ab-V378I mutation was highlighted, in which some infections may be associated with infections caused in the Middle East, including two Taiwanese (CGMH-CGU-10 and -11) strains having a travel history to Turkey, and some strains from Australia and Germany having a travel history to Iran. Currently, there are abundant viral genomic data from Asia, Europe, and America but very few from the Middle East. Our findings may contribute to developing a better understanding of the global SARS-CoV-2 transmission dynamics.

We detected a 382-nt deletion covering nearly the entire ORF8 of the genome of the CGMH-CGU-02 isolate obtained from a patient who returned from Wuhan in February 2020. A similar observation was found in Singapore strains [20]. These observations suggest the possible circulation of this strain in Wuhan in the first few months of the outbreak, as well as transmission to other geographical regions. Deletions in ORF8 were also observed during the SARS-CoV outbreak in 2003, which were associated with a reduced ability for virus replication in human cells [21]. The SARS-CoV-2 strains with this ORF8 deletion are believed to be still evolving and might have different evolutionary paths compared with those having no such deletion.

Despite the overall high sequence similarity, different clusters of SARS-CoV-2 strains identified in several countries can be distinguished based on phylogenetic analysis, and each cluster has been characterized by its conserved and unique mutations (Figure 3). It is not unusual to have viral mutations during an outbreak, especially for RNA viruses, but mutations that might affect virulence or pathogenicity are of concern. The second-wave strains circulating in Taiwan (Figure 2E and 3E) are characterized by Asp to Gly mutation at position 614 of the S gene that encodes the spike protein required for viral entrance. SARS-CoV-2 spike protein is divided into S1 and S2 subunits that function in receptor binding and membrane fusion, respectively [22]. Biochemical and structural studies have indicated several unique residues of SARS-CoV-2 that greatly improve the receptor-binding activity [23–26]. In addition, a four amino acid, Pro-Arg-Arg-Ala, inserted immediately upstream of the S1/S2 cleavage site generates a polybasic cleavage site of ubiquitous furin-like proteases that might affect the tissue tropism and/or transmissibility [22]. Lau et al. further used a Vero-E6 culture system to detect an attenuated variant with S1/S2 junction deletions [27]. Whether the receptor-binding activity or furin cleavage might be altered by D614G mutation remains to be investigated. Moreover, its nearby region contains several glycosylations as determined from the glycosylation shield structures [28]. Whether the mutation might change the glycosylation status and eventually change antigenicity awaits further analysis.

RNA viruses show variations in their genomes due to nucleotide substitutions generated by the low fidelity of RNA-dependent RNA polymerase during replication. Such genomic variations are believed to facilitate the successful adaption of the virus to various hosts. Previous studies show that the mutation rates of RNA viruses vary in different viruses and depend on the viral transmission modes [29]. Sequence analysis of SARS-CoV-2 isolated from 5 clustered infections between February 26 to March 9, 2020 in Taiwan revealed only 4 mutations in their 29,903-nt genomic RNA, suggesting that the nucleotide substitution rate was limited during viral RNA replication. The nsp14 exoribonuclease encoded by several coronaviruses plays a crucial role in proofreading during genome replication [30,31]. Investigation of the function of SARS-CoV-2 nsp14 and its replication fidelity is required to be undertaken in the future.

Metagenomic sequencing can help in rapidly exploring the genomic content of targeted viruses in a sample. It also helps in detecting any other pathogens in the microenvironment. *Haemophili* are common and representative bacterial species found in the upper respiratory tract samples of patients, and play an important role between the host and the environment [32,33]. Molyneaux et al. and Hofstra el al. found a significant outgrowth of *Haemophilus influenzae* and *Haemophilus parainfluenzae* from the pre-existing upper respiratory tract after a rhinovirus infection in subjects with chronic obstructive pulmonary disease and healthy volunteers, respectively [34,35]. Kosikowska et al. further demonstrated that *Haemophilus parainfluenzae* could be a marker of microbiota changes in the upper respiratory tract caused by antibiotics [36]. Ou et al. recently detected *Haemophilus parainfluenzae* and *Moraxella catarrhalis* from sputum samples collected from a severe COVID-19 case [37]. Although the prevalence and clinical impact of *Haemophilus parainfluenzae* in SARS-CoV-2-positive cases remain unclear, we demonstrated the advantage and capacity of metagenomic NGS in identifying co-existence with *Haemophilus parainfluenzae*. It suggested that further NGS studies are required to determine the respiratory microbiota composition and detect the co-infection/existence with other respiratory pathogens, providing new insights to the association between the virus with other pathogens.

In summary, two waves of the COVID-19 pandemic were documented in Taiwan. The first wave mostly included patients who had returned from China and the second one mostly included those who had travelled to Europe and Americas. We found a 382-nucleotide deletion in open reading frame 8 (ORF8) in one isolate in the first wave, as well as 5 clustered cases failed to trace to any imported ones and were hence considered sporadic local transmission cases. Moreover, these 5 genomes were phylogenetically designated to a clade harbouring the ORF1ab-V378I mutation, which is different from the 3 previously reported clades ORF8-L84S, ORF3a-G251V and S-D614G. Also included in this clade were some strains obtained from patients who had a travel history to Turkey and Iran. Highlighting this clade may provide important viral genome information regarding the COVID-19 outbreaks in the Middle East.

## Supplementary Material

Supplemental_Figure_S2-1_final.jpg

Supplemental_Figure_S1-1_final.jpg
