# Supplementary figures and images for: SARS-CoV-2 genomic surveillance in Taiwan revealed novel ORF8-deletion mutant and clade possibly associated with infections in Middle East
Source: Emerg Microbes Infect. 2020 Jul 3;9(1):1457–66. doi: 10.1080/22221751.2020.1782271 (PMC7473175; doi:10.1080/22221751.2020.1782271)

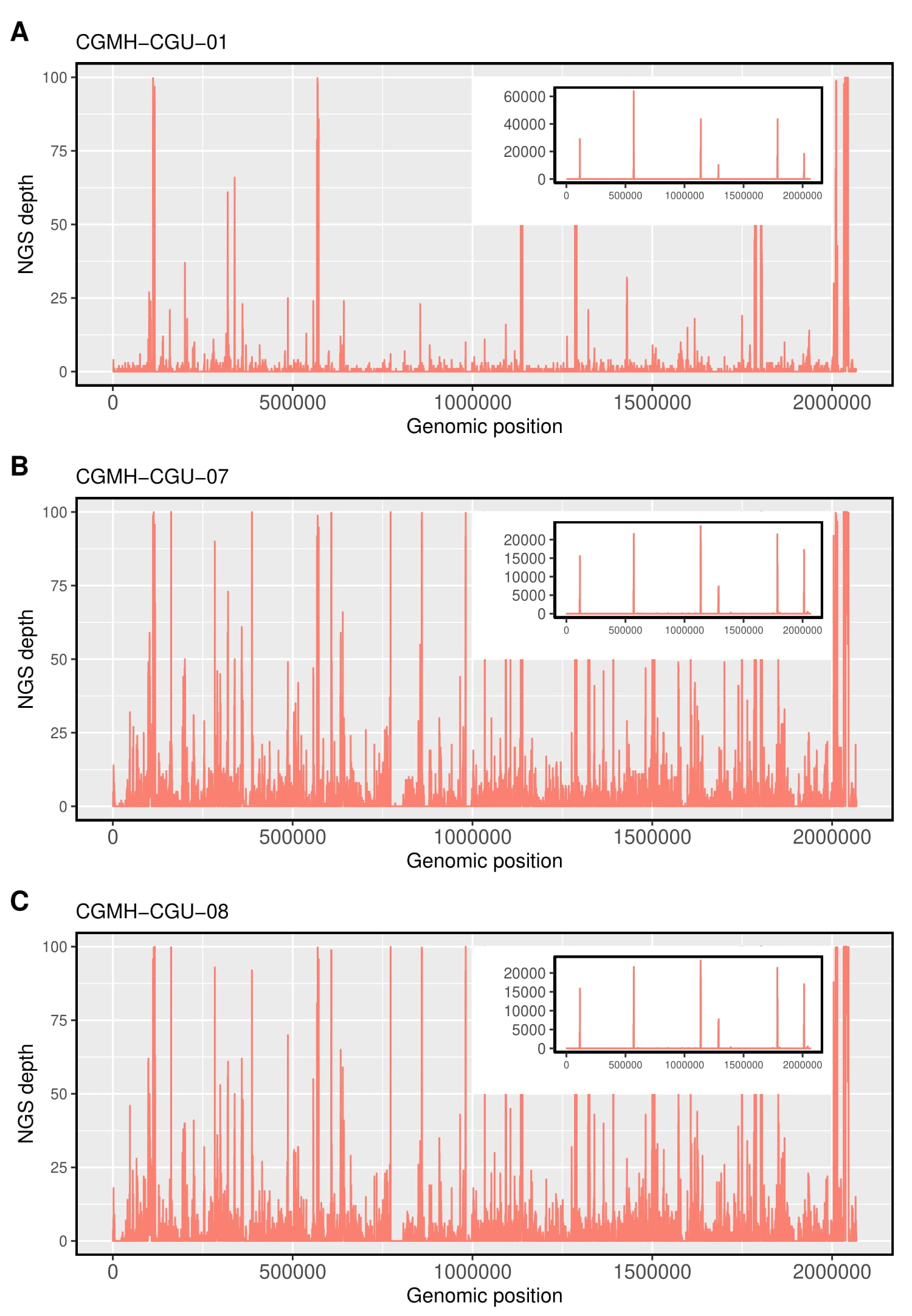

Supplement: Supplemental_Figure_S2-1_final.jpg [file TEMI_A_1782271_SM4509.jpg]

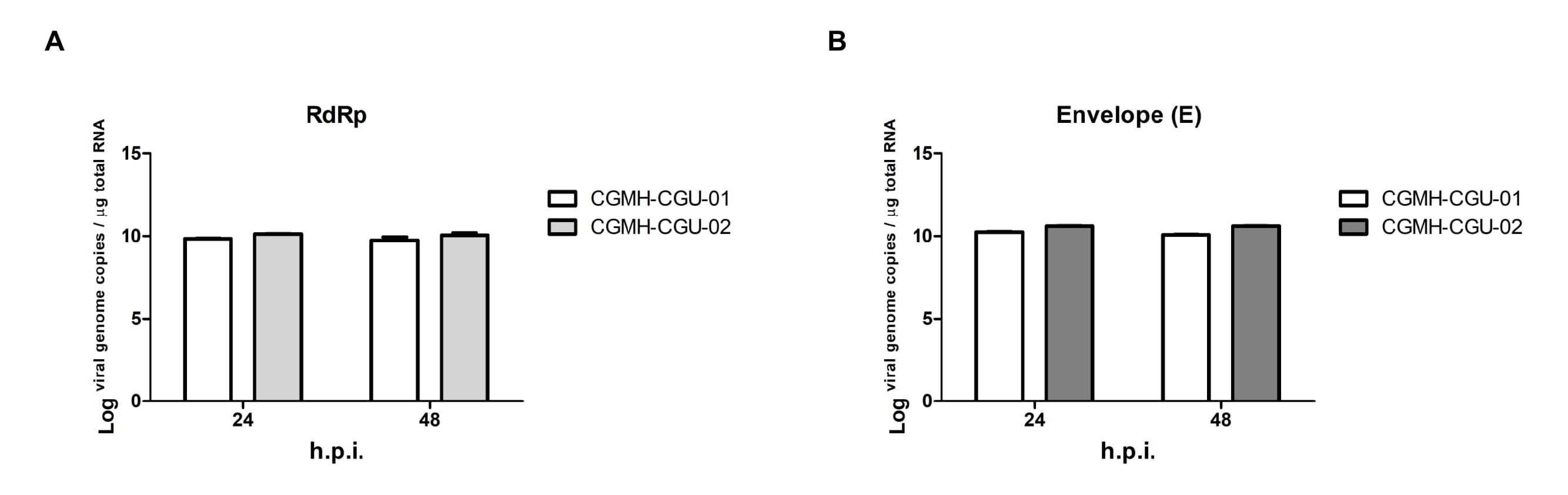

Supplement: Supplemental_Figure_S1-1_final.jpg [file TEMI_A_1782271_SM4508.jpg]
